# Advances of Techniques in Deep Regional Blocks

**DOI:** 10.1155/2017/7268308

**Published:** 2017-12-04

**Authors:** Jui-An Lin, Rafael Blanco, Yasuyuki Shibata, Tatsuo Nakamoto

**Affiliations:** ^1^Department of Anesthesiology, Wan Fang Hospital, Taipei Medical University, Taipei, Taiwan; ^2^Department of Anesthesiology, School of Medicine, College of Medicine, Taipei Medical University, Taipei, Taiwan; ^3^Department of Anesthesia, Corniche Hospital, Abu Dhabi, UAE; ^4^Department of Anesthesiology, Nagoya University Hospital, Nagoya, Japan; ^5^Department of Anesthesiology, Kansai Medical University, Hirakata, Japan

Advancing the techniques of deep regional blocks mainly relies on enhancing ergonomics and patient safety. As for ergonomics, efforts should be made to aid in decision-making process (cognitive ergonomics), to work with efficiency (e.g., shorter performance or onset time) and comfort (physical ergonomics), and to facilitate the operating room turnover by optimizing multidisciplinary cooperation (organizational ergonomics). Featured in this special issue is the updated reviews of several deep regional blocks on anatomy and techniques. Decision-making process (cognitive ergonomics) will be improved by the unified nomenclature system based on integrated applied anatomy and recommendation of suitable techniques for specific situations in these reviews. In the article reviewing obturator nerve block for this special issue, proximal approaches are recommended whenever possible, and the best patient position (supine or lithotomy) is yet to be determined. Although the authors worried about difficult alignment of the needle with the transducer in the supine position, needle visualization can be greatly facilitated by a true echogenic needle and meanwhile maintaining the eye, hand, needle, transducer, and ultrasound machine all in the same plane [1] according to recent advances in physical ergonomics [2, 3]. Furthermore, the supine position naturally keeps the leg straight and slightly externally rotated, which yields the best imaging on the obturator nerve [4]. To increase operating room efficiency (organizational ergonomics) for transurethral resection of bladder tumor, obturator nerve block is generally performed with the patient in the supine position immediately following spinal anesthesia, because preparation of lithotomy position (and subsequent surgical asepsis) following obturator nerve block could be viewed as a part of the interval for the deposited local anesthetic to take effect.

As for patient safety, ultrasound guidance for deep regional blocks is associated with significant limitations despite its popularity, which can be attributed to either the ultrasound machine (decreased ability to insonate deep neural structures) or the operator (errors in perception or interpretation in needle-nerve proximity during deep regional blocks) [5]. The inability of ultrasound to reliably insonate and locate deep neural structures could be circumvented with adjunctive neurostimulation. However, for some deep regional blocks (such as distal infraclavicular block [6, 7]), neurostimulation unnecessarily increases procedural time when there exist both a visible sonographic target and an identifiable sonographic endpoint. Thus, on the basis of ultrasound guidance as the gold standard, an important question has been raised regarding the definition of so-called “dual guidance” when choosing among ultrasound guidance, neurostimulation, and pressure monitoring for peripheral nerve blocks. Although ultrasound guidance indisputably leads to fewer needle passes for single-injection blocks, it seems to encourage small readjustment of needle tip position and multiple injections of local anesthetics [8, 9]. The ability of neurostimulation to predict relative needle-nerve proximity is not only limited by low sensitivity [10], but also attenuated by previous local anesthetic spread nearby [9, 11, 12]. Furthermore, current ultrasound technology may lack the resolution to differentiate epineurium from perineurium, and clinicians cannot always delineate cervical nerve roots clearly [12], let alone much deeper neural targets. These might explain, at least in part, the reason why the risk of neurological complications remains unchanged despite increased use of ultrasound guidance [13]. A 2015 consensus statement from the American Society of Regional Anesthesia and Pain Medicine expanded the recommendations to apply injection pressure monitoring for earlier detection of needle-nerve contact and avoidance of intrafascicular injection during peripheral nerve blocks [14]. Detection of needle-nerve contact on both oligofascicular [12] and multifascicular [11] neural targets can be greatly enhanced by monitoring opening injection pressure, which is the pressure level that must be overcome to initiate injection into the target place [15]. As for prevention of intrafascicular spread, pressure monitoring may prove to be most useful for its negative predictive value as no cases have been reported to suffer from functional neuropathy with low injection pressures [10, 16]. Because opening injection pressure of more than 20 psi is associated with intrafascicular spread, 15 psi has been chosen as the cut-off pressure to keep 5 psi lower than the lowest reported value that resulted in neurological injury [11, 12]. Clinically at this cut-off point, high opening injection pressure (>15 psi) can consistently detect needle-nerve contact [12], indicating that a typical perineural injection requires low opening pressure (<15 psi) [10]. This cut-off point (15 psi) was further supported by a fresh human cadaver model, where opening injection pressure for intraneural injection was ranging from 21.5 ± 4.9 psi to 25.8 ± 4.3 psi for common ultrasound-guided lower extremity blocks [17]. A volume of 5 mL injection into the brachial plexus root within 15 sec results in a much higher opening injection pressure (>30 psi) in 100% fresh cadavers [18]. A human study demonstrated cessation of injection when opening pressure more than 20 psi for popliteal sciatic block does not result in neurological dysfunction [19].

Although several means of monitoring injection pressure have been recommended [20, 21], its popularization is limited by the facilities required (e.g., the pump or the commercial kits) until the advent of a convenient alternative utilizing the half-the-air setting [9]. The half-the-air setting consists of a central stopcock with its male luer lock connecting an extension tube to the patient, a side female luer lock to the 5% dextrose water (D5W) syringe, and an end female luer lock to the local anesthetic syringe [9]. The concept of “half-the-air” to keep injection pressure below 15 psi derives from the reliability of compressed air injection technique [22, 23], and the facility-independent half-the-air setting helps popularize injection pressure monitoring in clinical practice by the advantages of low cost, easy assembling, and incorporating a test syringe (ensuring the total mass of local anesthetic to be delivered) and a long (200 cm) extension tube (facilitating the operation for the assistant, thus physical ergonomics) with a low dead space (1.4 mL) [9]. Thereafter, another improvised pressure gauge was proposed using the fluid meniscus level in the 1 ml syringe in place of D5W syringe as the passive indirect in-line manometer [24]. However, as with the drawback of the commercial in-line manometer, this improvised pressure gauge lacks a pop-off valve to limit the injection pressure and/or eliminate the initial high peak pressure [20, 24]. In other words, with the in-line manometer only as a monitor, “syringe feel” is still performed at the very beginning of injection. It would be safer to limit the initial pressure inherent to the act of half-the-air [9, 23] rather than only an in-line pressure gauge without a physical pressure limit. Theories behind the half-the-air setting are based on simple rules of physics. According to Pascal's law, pressure within the syringe, tubing, and needle is equal throughout the system until the opening pressure is reached (and flow begins to occur), regardless of the speed of injection, the lumen size, or the size of fluid passage [11, 12]. Consequently, the syringe pressure could accurately reflect the needle tip pressure in the closed system [15]. The flow through the needle tip will start upon reaching the opening pressure of the target tissue. In this dynamic phase, the needle tip pressure will be kept below the original exerted pressure within the syringe because of two reasons. First, as the fluid accelerates through a constriction of the needle, the pressure reduces owing to the Bernoulli effect [20]; second, according to the fact that, with the needle tip open to the atmosphere, the speed of the injectate coming out of the needle tip declines shortly after commencing the injection by the act of half-the-air, we can confirm that the injection pressure declines following air compression to a given volume during the same course of half-the-air technique. If flow stops during drug administration, it would reliably reflect syringe pressure in response to accumulated volume of the injectate within the tissue, because, again by Pascal's law, the injection pressure returns to a state in which the pressures transmitted are equally distributed throughout the closed system [15]. Expert opinion shows that as the evidence for the utility of injection pressure as a safety monitor continues to accumulate, injection pressure monitoring should be incorporated as the routine during peripheral nerve blocks as long as the setting is not expensive and easy to apply [25]. From what was described above, pressure monitoring in combination with ultrasound guidance should be regarded as what we called “dual guidance” for deep regional blocks. Otherwise, hydrodissection by D5W at all times towards deep neural targets helps push away nerve fibers and minimize discomfort during ultrasound-guided needle advancement, as proposed in a research article regarding chronic pain management in this special issue.

The importance of half-the-air setting in deep regional blocks needs to be stressed beyond neurological complications. Ultrasound guidance unequivocally decreases the incidence of local anesthetic systemic toxicity [26]. However, it seems that the benefit cannot apply to deep regional blocks because of the paradox inherent to ultrasound guidance for deeper targets [5]. A dose-finding study demonstrated that Shamrock method for lumbar plexus block, the archetype of deep regional blocks, “might improve but not eliminate all challenges related to a deep ultrasound-guided nerve block” [27], and complications encountered in their study include local anesthetic systemic toxicity and epidural spread. The authors claimed that systemic toxicity might result from a needle tip partially placed inside a blood vessel, although local anesthetic spread was observed during injection [27]. Probably before the spread is visible, significant amounts of local anesthetic have already been injected into the vessels [28]. This assumption urges, in addition to ultrasound guidance and neurostimulation, the need for incorporating the half-the-air setting to minimize local anesthetic systemic toxicity during lumbar plexus block [28, 29] because the volume consumed to hydrolocate the needle tip and to visualize the hypoechoic spread is D5W instead of local anesthetic [9]. As for epidural spread during lumbar plexus block, it is not the injection volume but the injection pressure that matters [28, 30]. Although the original half-the-air setting [9] can be used to check the initial pressure prior to injection of local anesthetic (ensuring opening injection pressure <15 psi), modification of the setting has been urged to avoid pressure fluctuation during local anesthetic injection [31]. In response to the request, performing the same half-the-air technique in the local anesthetic syringe after D5W spread may keep the injection pressure at (when the liquid level starts to decrease) or below (while liquid level is descending) 14.7 psi throughout. Our preliminary qualitative analysis demonstrated that, by using the pressure management system of Injectomat Agilia® pump (Fresenius Vial, Brezins, France) as an in-line manometer between the needle (the tip inserted 3 cm into the pork model) and the low-dead space extension tube, pushing pressure generated by the act of half-the-air was below 15 psi during injection (experiment was run in triplicate, and occlusion alarm did not occur after the flow had commenced in response to half-the-air pressure exerted in the 20 mL local anesthetic syringe with the syringe pump set to an infusion rate of 0.1 ml/h and a pressure limit of 750 mmHg). Therefore, to improve all aspects at the same time for lumbar plexus block [28, 29], the modified half-the-air setting (additionally adding the act of half-the-air in the local anesthetic syringe) might reduce epidural spread (half-the-air in both the D5W and local anesthetic syringes) and local anesthetic systemic toxicity (D5W test spread in response to half-the-air) as well as nerve injury (half-the-air in the D5W syringe) during lumbar plexus block. Utilizing a syringe with a larger capacity (such as 25 or 30 mL) for the modified half-the-air setting will increase the drug volume to be administered in a single syringe if the practitioner standardizes the air volume compressed (5 mL) in the local anesthetic syringe with 10 mL of air introduced above the fluid. To more easily administer the intended local anesthetic volume by maintaining a higher but no more than 15 psi pushing pressure, repeating the act of half-the-air could help empty the local anesthetic syringe without delay. As with the original one [28], D5W emptied in the low dead space (1.4 ml) extension tube before local anesthetic injection in the modified half-the-air setting will not result in a significant dilutional effect for lumbar plexus block.

Some deep regional blocks require precise hydrodissection of a specific fascial or interfascial plane. A test spread other than local anesthetic from the half-the-air setting [9] helps identify the correct target without consumption of local anesthetic in the wrong place or the need of adding total mass (thus toxicity) to achieve the desired effect, as mentioned in the review articles on transversus abdominis plane block and obturator nerve block for this special issue. Furthermore, a retrospective study from the same special issue indicates that D5W itself is a potential analgesic for hydrodissection. Compared with normal saline, D5W also preserves neurostimulation for deep regional blocks when needed. Therefore, D5W is the test solution of choice in the half-the-air setting. Compared to pure pressure monitors, the half-the-air setting is not only multifunctional but also a safer pressure limiter inherent to the act of half-the-air. In the absence of other advanced monitoring techniques, there is no reason to perform ultrasound-guided deep regional blocks without incorporating the half-the-air setting, complying with the recommendations from the review articles in this special issue.
