# Corrigendum to “Advances of Techniques in Deep Regional Blocks”

**DOI:** 10.1155/2018/5151645

**Published:** 2018-07-05

**Authors:** Jui-An Lin, Rafael Blanco, Yasuyuki Shibata, Tatsuo Nakamoto, Ko-Huan Lin

**Affiliations:** ^1^Department of Anesthesiology, Wan Fang Hospital, Taipei Medical University, Taipei, Taiwan; ^2^Department of Anesthesiology, School of Medicine, College of Medicine, Taipei Medical University, Taipei, Taiwan; ^3^Department of Anesthesia, Corniche Hospital, Abu Dhabi, UAE; ^4^Department of Anesthesiology, Nagoya University Hospital, Nagoya, Japan; ^5^Department of Anesthesiology, Kansai Medical University, Hirakata, Japan; ^6^Division of Psychiatry, Hualien Armed Forces General Hospital, Hualien, Taiwan

In the article titled “Advances of Techniques in Deep Regional Blocks” [1], Table 1 was missing. Accordingly, the table is shown below, and its in-text citation is added as follows:

Our preliminary qualitative analysis demonstrated that, by using the pressure management system of Injectomat Agilia® pump (Fresenius Vial, Brezins, France) as an in-line manometer between the needle (the tip inserted 3 cm into the pork model) and the low-dead space extension tube, pushing pressure generated by the act of half-the-air was below 15 psi during injection (experiment was run in triplicate, and occlusion alarm did not occur after the flow had commenced in response to half-the-air pressure exerted in the 20 mL local anesthetic syringe with the syringe pump set to an infusion rate of 0.1 ml/h and a pressure limit of 750 mmHg) (Table 1).

## Figures and Tables

**Table 1:** Qualitative analysis of injection pressure for the half-the-air setting.

Compressed air volume	50% (half-the-air) (5 ml compression)			60% (6 ml compression)		
Repeated experiments	Set 1	Set 2	Set 3	Set 1	Set 2	Set 3
Step 1: not open to the needle						
						
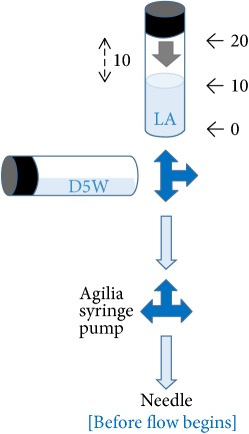	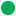	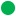	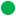	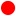	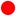	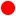

Step 2: maintaining step 1 pressure and then open to the needle						
				
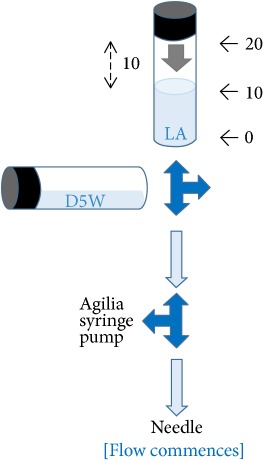	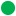	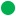	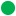	Unable to assess the pressure by alarm in a consecutive (step 1 and then step 2) manner. (already alarmed in step 1)		

By using the pressure management system of Injectomat Agilia pump (Fresenius Vial, Brezins, France) as an in-line manometer, pressure within the half-the-air setting (ten milliliters of air was aspirated into the syringe above 10 ml local anesthetic [LA]) was assessed by adding an extra three-way stopcock between the needle (the tip inserted 3 cm into the pork) and the low-dead space extension tube with the side female luer lock connecting to the Agilia syringe pump via a pressure tube. The syringe pump was set to a minimal infusion rate of 0.1 ml/h and a pressure limit of 750 mmHg. Each experiment was run in triplicate. Green dot: “without” pressure (occlusion) alarm within 5 sec of injection. Red dot: “with” pressure (occlusion) alarm within 5 sec of injection. D5W: 5% dextrose water.

## References

[1] Lin J.-A., Blanco R., Shibata Y., Nakamoto T. (2017). Advances of techniques in deep regional blocks. *BioMed Research International*.

